# Importance of beta cell mass for glycaemic control in people with type 1 diabetes

**DOI:** 10.1007/s00125-022-05830-2

**Published:** 2022-11-17

**Authors:** Theodorus J. P. Jansen, Maarten Brom, Marti Boss, Mijke Buitinga, Cees J. Tack, Lian A. van Meijel, Bastiaan E. de Galan, Martin Gotthardt

**Affiliations:** 1 Department of Medical Imaging, Radboud University Medical Center, Nijmegen, the Netherlands; 2 Nutrition and Movement Sciences, Maastricht University, Maastricht, the Netherlands; 3 Radiology and Nuclear Medicine, Maastricht UMC+, Maastricht, the Netherlands; 4 Internal Medicine, Radboud University Medical Center, Nijmegen, the Netherlands; 5 Internal Medicine, Maxima Medical Center, Veldhoven, the Netherlands; 6 Internal Medicine, Maastricht UMC+, Maastricht, the Netherlands; 7 CARIM School for Cardiovascular Disease, Maastricht University, Maastricht, the Netherlands

**Keywords:** Beta cell mass, Exendin, Glucose control, Glucose variability, Hypoglycaemic burden, Pancreatic beta cell, PET imaging, Type 1 diabetes

## Abstract

**Aims/hypothesis:**

The role of beta cell mass in the balance of glucose control and hypoglycaemic burden in people with type 1 diabetes is unclear. We applied positron emission tomography (PET) imaging with radiolabelled exendin to compare beta cell mass among people with type 1 diabetes and either low glucose variability (LGV) or high glucose variability (HGV).

**Methods:**

All participants with either LGV (*n*=9) or HGV (*n*=7) underwent a mixed-meal tolerance test to determine beta cell function and wore a blinded continuous glucose monitor for a week. After an i.v. injection with [^68^Ga]Ga-NODAGA-exendin-4, PET images were acquired for the quantification of pancreatic uptake of radiolabelled exendin. The mean standardised uptake value (SUVmean) of the pancreas was used to determine the amount of beta cell mass.

**Results:**

Participants with LGV had lower HbA_1c_ (46.0 mmol/mol [44.5–52.5] [6.4% (6.3–7)] vs 80 mmol/mol [69.0–110] [9.5% (8.5–12.2)], *p*=0.001) and higher time in range (TIR) (75.6% [73.5–90.3] vs 38.7% [25.1–48.5], *p*=0.002) than those with HGV. The SUVmean of the pancreas was higher for the LGV than for the HGV group (5.1 [3.6–5.6] vs 2.9 [2.1–3.4], *p*=0.008). The AUC_C-peptide_:AUC_glucose_ ratio was numerically, but not statistically, higher in the LGV compared with the HGV group (2.7×10^−2^ [6.2×10^−4^–5.3×10^−2^] vs 9.3×10^−4^ [4.7×10^−4^–5.2×10^−3^], *p*=0.21). SUVmean correlated with the AUC_C-peptide_:AUC_glucose_ ratio (Pearson *r*=0.64, *p*=0.01), as well as with the TIR (*r*=0.64, *p*=0.01) and the SD of interstitial glucose levels (*r*=−0.66, *p*=0.007).

**Conclusion/interpretation:**

Our data show higher beta cell mass in people with type 1 diabetes and LGV than in those with HGV, independent of beta cell function.

**Graphical abstract:**

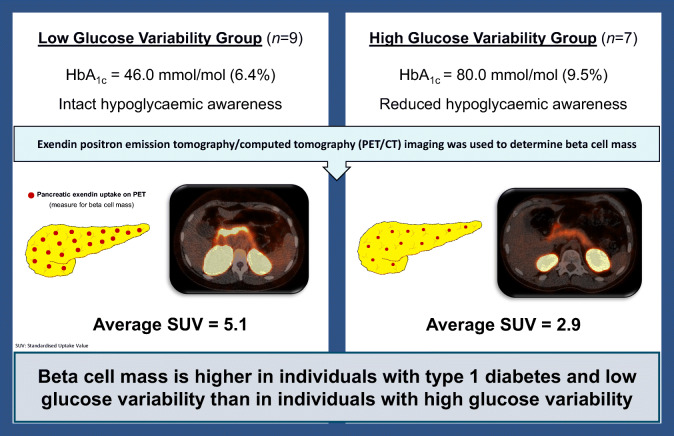

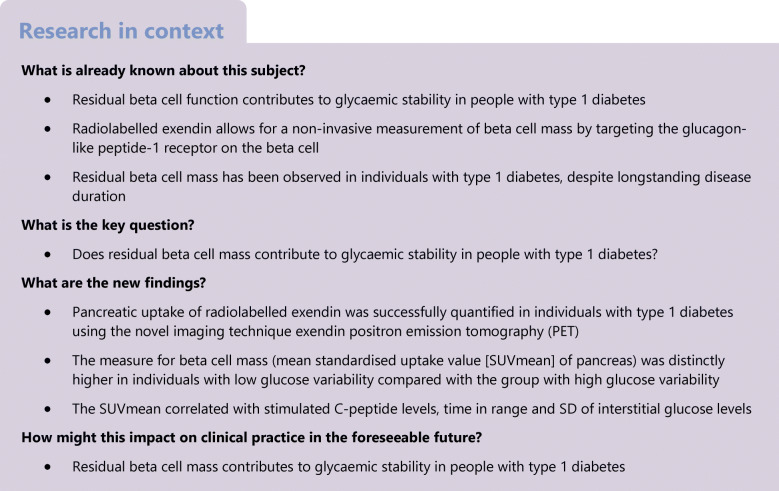



## Introduction

Despite widespread implementation of basal-bolus insulin regimens as the most adequate treatment for type 1 diabetes, there are large interindividual differences in the level of glycaemic control. Only a minority of people with type 1 diabetes achieve widely accepted treatment targets [1], which has been attributed to increased risks of hypoglycaemia associated with optimisation of glucose control. Remarkably, however, some individuals seem to have achieved HbA_1c_ at or below target levels without a high hypoglycaemic burden, whereas others with much poorer glucose control suffer from frequent hypoglycaemia, including severe episodes, and considerable high glucose variability (HGV). Although certain psychological and behavioural factors, such as fear and consequent avoidance of hypoglycaemia [2] may explain some of these disparities, the underlying mechanisms remain largely unknown.

The loss of insulin production capacity in type 1 diabetes has long been considered the inevitable consequence of the complete destruction of pancreatic beta cells through a targeted cytotoxic autoimmune attack [3]. Beta cells that escape this autoimmune attack and retain functional capacity, clinically reflected by low but detectable C-peptide levels and insulin-positive immunohistochemical analysis [4–11], appear to have a positive effect on glucose variability, hypoglycaemic burden and overall glycaemic control [12, 13]. Cumulative research shows that even in longstanding type 1 diabetes, a considerable number of beta cells can survive the immune attack, even in the absence of retaining beta cell function [5–7].

Recent advances in the field of in vivo quantification of beta cells allow us to monitor beta cell mass non-invasively. Currently, the best characterised and most specific tracer to visualise beta cells in vivo is radiolabelled exendin, binding to the glucagon-like peptide 1 (GLP-1) receptor on the beta cell [14–18]. Indeed, we showed persistent beta cell mass in people with longstanding type 1 diabetes using single photon emission computed tomography (SPECT) imaging [17]. To what extent such residual beta cell mass contributes to the level of glucose stability in people with type 1 diabetes is currently unknown. To examine this further, we applied the more accurate imaging technique positron emission tomography (PET) with radiolabelled exendin [19], for non-invasive quantification of beta cell mass in individuals with type 1 diabetes and glucose profiles with either low or high variability.

## Research design and methods

### Study participants

All study participants were recruited from the diabetes outpatient clinic of the Radboud University Medical Center (Nijmegen, the Netherlands) and through online advertisements. All individuals had to have had type 1 diabetes for at least 1 year.

Inclusion criteria for the low glucose variability (LGV) group included an HbA_1c_ of ≤53 mmol/mol (≤7%), intact hypoglycaemic awareness (as defined by a modified Clarke score of 0 or 1 [20]) and no experience of severe hypoglycaemia, defined as an event requiring assistance from another person to recover [21], in the past year and no more than two severe events overall.

The HGV group complied with the following criteria: either an HbA_1c_ of ≥69 mmol/mol (≥8.5%) with reduced hypoglycaemic awareness (modified Clarke score of ≥2) and/or at least two severe hypoglycaemic events in the past year, or an HbA_1c_ of ≥64 mmol/mol (≥8.0%) with impaired awareness of hypoglycaemia (modified Clarke score of ≥3) and/or at least two severe hypoglycaemic events in the past year.

All study procedures were performed at the Radboud University Medical Center. This clinical study was approved by the local Institutional Ethics Review Committee and study participants gave written informed consent before participation (ClinicalTrials.gov number: NCT03785275).

### Mixed-meal tolerance test

All individuals underwent a mixed-meal tolerance test (MMTT) to assess their beta cell function [22]. The MMTT was performed in the morning, preceded by a 12 h fasting period, during which only water was consumed. Participants were asked to abstain from using short-acting insulin for 6 h before the test and to reduce the dose of long-acting insulin or the basal rate of their insulin pump by 30–35% on the preceding day. Before the MMTT, blood was drawn to determine fasting glucose, C-peptide and insulin. Additionally, blood samples were taken to measure HbA_1c_ and to assess kidney function and liver enzymes. Subsequently, participants consumed 6 ml/kg liquid meal (Nutridrink, Nutricia, the Netherlands) to a maximum of 360 ml within 5 min. Blood samples were collected at 0, 15, 30, 60, 90 and 120 min after ingestion to determine stimulated glucose, C-peptide and insulin levels. The detection limit of the C-peptide assay was 0.01 nmol/l and in case of unmeasurable low C-peptide, 0.01 nmol/l was noted as measured value for that specific timepoint.

AUC for basal and stimulated glucose and C-peptide were calculated using Prism 5.03 software (GraphPad Software, San Diego, CA, USA). To determine a better estimate of the residual beta cell function than the AUC for C-peptide alone, the AUC_C-peptide_:AUC_glucose_ ratio was calculated. These AUC values, peak C-peptide measurements and AUC_C-peptide_:AUC_glucose_ ratios were correlated with the results of the image analysis.

### Continuous glucose monitoring

All participants were asked to wear a blinded glucose sensor for continuous glucose monitoring (CGM) (Dexcom G4 or G6, Dexcom, San Diego, CA, USA). The Dexcom G4 system was used in the first 14 individuals and the Dexcom G6 system in the last two participants after the transition to this new CGM system. CGM data of the first participant were not available at the time of analysis. The glucose sensor for the study was placed after the MMTT to measure for a period of 7 to 8 days (depending on scan date) and was removed prior to the PET/computed tomography (CT) scan. Glycaemic variables were based on glucose measurements starting on the day after the MMTT and excluding the day of the PET/CT acquisition (because of the fasting period). The glycaemic variables included mean glucose levels with their corresponding SD and CV as measure for glycaemic variability. Furthermore, the percentage of time that glucose levels were in range (TIR, glucose 3.9 to 10.0 mmol/l), below range (TBR, <3.9 mmol/l) and above range (TAR, >10 mmol/l) were obtained.

### PET/CT acquisition

Image acquisition was performed using a Siemens Biograph 40 mCT time-of-flight PET/CT system. Prior to PET/CT imaging, study participants fasted for at least 4 h to prevent interference with endogenous GLP-1 production, and insulin use was temporarily adjusted in the same manner as was done prior to the MMTT. Blood samples were drawn just prior to image acquisition to determine blood glucose levels.

Static PET images were acquired 60 min after a slow i.v. bolus injection of 76.6±2.9 MBq [^68^Ga]Ga-NODAGA-exendin-4 (peptide dose 3–7 μg), further referred to as exendin PET. Radiochemical preparation was done as previously described [14]. Image data were obtained with two bed positions (10 min/bed position) of the abdominal region with the pancreas in the field of view. For anatomical information and attenuation correction, a low-dose CT scan without contrast of the abdomen was acquired. The CT transaxial matrix was 512×512 (0.98×0.98 mm) with a CT slice width of 3 mm. The PET data were reconstructed with three iterations, 21 subsets and a post-reconstruction Gaussian filter of 3 mm full width at half maximum.

### Quantitative image analysis

The reconstructed PET/CT images were analysed using Inveon Research Workplace 4.1 software (Siemens Healthcare, Erlangen, Germany). Volumes of interest (VOIs) were drawn around the pancreas, duodenum and kidneys, based on the CT images. Radiolabelled exendin is cleared via the kidneys which results in high renal uptake. The kidney VOIs were dilated by 6 mm to include all renal radioactivity. Then the pancreas VOIs were corrected for the spill-over from the kidneys by excluding the activity originating from the kidney VOIs. This prevents overestimation of the pancreatic uptake resulting from the closely located left kidney. In addition, the pancreatic head is in some cases situated nearby the duodenum. Therefore, the activity that originated from the duodenal VOIs was also excluded from the VOIs of the pancreas in a similar manner as for the kidneys.

The quantification of the PET/CT data provided mean uptake values (Bq/ml). By correcting for injected activity and body weight, the mean standardised uptake value (SUVmean [unitless]) of the pancreas was determined. The SUVmean allows for a reliable comparison between individuals and patient populations with different characteristics (e.g. groups with LGV and HGV). The SUVmean of the pancreas was therefore used as measure for (residual) beta cell mass.

### Statistical analysis

The sample size was determined using the data acquired in a previous study with exendin SPECT [17]. This resulted in a sample size of 18 participants, nine in each group (significance level of 0.05 and power of 0.8). Seven individuals were included in the HGV group due to difficulties in recruitment caused by the COVID-19 pandemic, strict inclusion criteria and burdensome study protocol. However, this was thought acceptable given that the better spatial resolution and improved image quantification of PET would allow for better detection of small differences in pancreatic uptake compared with SPECT.

Acquired data were expressed as mean±SD, median (IQR), or number (%). The Mann–Whitney *U* test was used to assess group differences. Relationships between variables were checked for linearity using the Pearson correlation coefficient (*r*), with a two-tailed ANOVA. The level of significance was set at *p*<0.05. GraphPad Prism software was used for all analyses (GraphPad Prism 5 for Windows).

## Results

We recruited nine participants to the LGV group (seven women) and seven participants to the HGV group (five men). Apart from the imbalance in women/men, the two groups did not differ with respect to age, BMI or diabetes duration (Table 1). In concordance with the protocol, HbA_1c_ and modified Clarke scores were lower in the LGV group (Table 1). Stimulated C-peptide levels were detectable in half of all individuals, but with no differences between the groups (56% vs 43%, *p*=0.21). Following injection of the radiotracer, two patients experienced nausea and one vomited, which are known side effects of exendin. No other adverse effects were observed.

**Table 1:** Clinical characteristics

Variables	LGV group (*n*=9)	HGV group (*n*=7)
Age (years)	40.2±16.1	36.9±18.9
Sex (female/male)	7/2	2/5
T1D duration (years)	13.8 (3.0–28.0)	13.0 (3.8–38.0)
HbA_1c_ (mmol/mol)	46.0 (44.5–52.5)	80.0 (69.0–110.0)*
HbA_1c_ (%)	6.4 (6.3–7.0)	9.5 (8.5–12.2)*
BMI (kg/m^2^)	23.9±3.3	25.5±2.7
CKD-EPI-GFR (ml/min per 1.73 m^2^)	87.3±8.0	89.0±2.6
Score on modified Clarke questionnaire	0 (0–1)	3 (2–3)*
Severe hypoglycaemic event in past year (events per individual)	0	0.29
Severe hypoglycaemic event throughout life (events per individual)	0.11	1.29

Data are shown as mean±SD, median (IQR) or number

*p<0.05 vs LGV group

CKD-EPI-GFR, Chronic Kidney Disease Epidemiology Collaboration glomerular filtration rate; T1D, type 1 diabetes

**Fig. 1:**
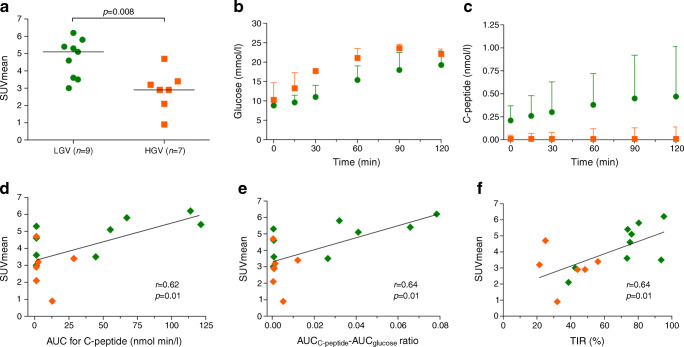
Analysis of MMTT and PET/CT data. SUVmean of the pancreas (**a**) in individuals with LGV (green) and HGV (orange). Glucose (**b**) and C-peptide profiles (**c**) for both groups, median (IQR). Correlations of the SUVmean with the AUC for C-peptide (**d**), the AUC_C-peptide_:AUC_glucose_ ratio (**e**) and with the percentage TIR (**f**). Two participants from the HGV group have a SUVmean of 2.90 and no detectable C-peptide and their data points are in the same location (**d** and **e**). CGM data of one participant from the HGV group were not available at the time of analysis and only six data points from this group are visualised (**f**)

Quantification of the PET/CT images revealed that pancreatic exendin uptake was distinctly higher in the LGV group as compared with the HGV group (Fig. 1a). This was the case for both the mean uptake value (2.5 [2.1–3.2] vs 1.5 [1.0–2.0] kBq/ml, *p*=0.005) and SUVmean of the pancreas (5.1 [3.6–5.6] vs 2.9 [2.1–3.4], *p*=0.008).

In the MMTT, glucose and C-peptide levels were measured (Fig. 1b and c). Variables of stimulated C-peptide levels were numerically higher in the LGV compared with the HGV group, but these differences did not reach statistical significance (AUC for C-peptide, 44.6 [1.2–90.6] vs 1.2 [1.2–12.8] nmol min/l [*p*=0.21] and peak C-peptide level, 0.47 [0.01–1.0] vs 0.01 [0.01–0.14] nmol/l [*p*=0.21]). Neither BMI nor diabetes duration correlated with the AUC for C-peptide. The AUC_C-peptide_:AUC_glucose_ ratio was also determined but was not different between the groups (2.7×10^−2^ [6.2×10^−4^–5.3×10^−2^] vs 9.3×10^−4^ [4.7×10^−4^–5.2×10^−3^], *p*=0.21).

The analysis of CGM data demonstrated that people in the LGV group had significantly higher TIR and lower mean glucose levels than those in the HGV group (Table 2). Also, both the SD of mean interstitial glucose levels and TAR were higher in the HGV group (Table 2), with no significant difference with respect to TBR between the groups (Table 2).

**Table 2:** Data from CGM

Variables	LGV group (*n*=9)	HGV group (*n*=7)
Mean glucose (mmol/l)	7.2 (6.3–7.9)	10.5 (10.3–12.6)*
SD (mmol/l)	2.1 (1.5–2.7)	4.4 (3.8–4.5)*
CV (%)	31.1 (22.6–36.1)	36.3 (34.8–43.4)
TIR (%)		
3.9–10.0 mmol/l	75.6 (73.5–90.3)	38.7 (25.1–48.5)*
TBR (%)		
<3.0 mmol/l	3.6 (0.1–6.7)	0.2 (0.0–2.0)
<3.9 mmol/l	6.6 (1.0–16.7)	1.9 (0.1–6.8)
TAR (%)		
>10 mmol/l	8.4 (4.4–19.8)	53.9 (47.3–66.8)*
>13.9 mmol/l	0.2 (0.0–2.7)	21.3 (18.9–39.8)*

Data are expressed as median (IQR)

*p<0.05 vs LGV group

**Fig. 2:**
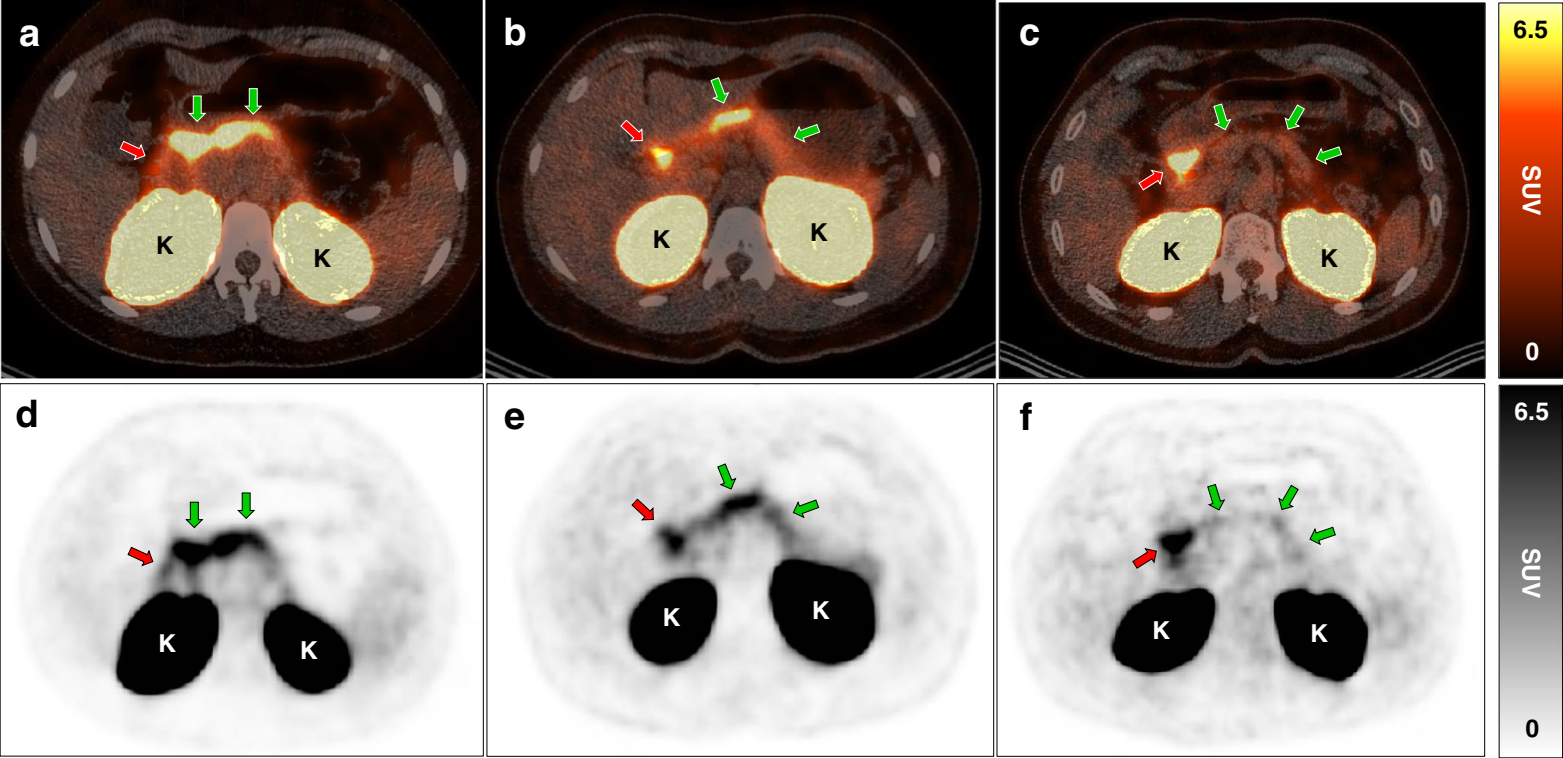
Abdominal PET/CT images with pancreatic uptake of radiolabelled exendin. Transversal fused PET/CT images (**a**–**c**) and PET images (**d**–**f**) of three individuals showing pancreatic uptake of ^68^Ga-exendin as measure for beta cell mass (green arrows). Other regions with exendin uptake are the proximal duodenum (red arrows) and the kidneys (indicated with the letter ‘K’). Pancreatic exendin uptake of individuals with LGV were in the same range for individual 1 (**a**, **d**) (AUC for C-peptide 122 nmol min/l) and individual 2 (**b**, **e**) (no detectable C-peptide), despite differences in C-peptide response, and much greater than in individual 3 (**c**, **f**) (no detectable C-peptide)

The uptake of exendin in the pancreas correlated to beta cell function (expressed as stimulated C-peptide), as reflected by correlations between the SUVmean and the AUC for C-peptide (Pearson *r*=0.62, *p*=0.01, Fig. 1d), peak C-peptide value (Pearson *r*=0.65, *p*=0.007), and AUC_C-peptide_:AUC_glucose_ ratio (Pearson *r*=0.64, *p*=0.01, Fig. 1e). Nevertheless, one of the individuals with LGV and no detectable C-peptide had a similar SUVmean as the individual with the highest stimulated C-peptide, which was much greater than in another individual without detectable C-peptide (Fig. 2). Furthermore, SUVmean correlated with TIR (Pearson *r*=0.64, *p*=0.01, Fig. 1f) and was inversely correlated with the mean glucose levels (Pearson *r*=−0.59, *p*=0.02), SD of glucose levels (Pearson *r*=−0.66, *p*=0.007) and TAR (Pearson *r*=−0.64, *p*=0.01). We observed no correlations between the SUVmean of the pancreas and BMI, diabetes duration, age at disease onset and blood glucose levels prior to imaging.

## Discussion

The main findings of this study are that beta cell mass as quantified by exendin PET was higher in people with type 1 diabetes and LGV compared with those with HGV. Multiple correlations were found between glycaemic variables and beta cell mass, pointing towards the importance of residual beta cells for the level of glycaemic control. Altogether, these results strongly support that preservation of beta cell mass additionally benefits glycaemic stability in people with type 1 diabetes alongside beta cell function.

In spite of historical belief, there is increasing evidence for beta cell survival in longstanding type 1 diabetes, substantiated by the presence of insulin-positive residual beta cells, and measurable levels of C-peptide and proinsulin [4–11, 23–25]. Using the novel imaging technique exendin PET, relevant pancreatic uptake of radiolabelled exendin was found in most study participants, consistent with the presence of residual beta cell mass, despite diabetes durations ranging from 2 to 50 years. This shows that beta cells can survive many years after the onset of diabetes. The greater exendin uptake in the LGV compared with the HGV group may underscore the importance of residual beta cell mass for glycaemic variables from a clinical point of view. Although residual beta cell function, as reflected by measurable C-peptide during the MMTT, may have contributed to better glycaemic stability [12, 26, 27], it is unlikely that this explains all the benefits. Indeed, observations of LGV in participants with high beta cell mass, despite undetectable C-peptide levels, argue for a beneficial role of beta cell mass, independent of beta cell function.

This disconnection between beta cell mass and function is best illustrated by two individuals with similar beta cell mass, one of whom displayed relatively high residual C-peptide production, whereas it was unmeasurable in the other (Fig. 2). The presence of beta cell mass thus does not necessarily mean that these residual beta cells are insulin-positive, let alone insulin-producing [5–7], while being positive for the GLP-1 receptor, yet the association with glycaemic variables suggests some functionality. A possible explanation may be suppression of glucagon release from alpha cells by residual beta cells [28] or a lower state of inflammation, both of which may allow for better glucose control. Future studies should consider also measuring glucagon, high-sensitivity C-reactive protein and cyclic citrullinated peptide antibodies to obtain a better understanding of this observation. The amount of beta cell mass, its potentially beneficial effect on glucose outcomes and the mechanisms behind this beneficial effect should be studied more extensively to understand the clinical significance of residual beta cell mass.

Radiolabelled exendin is so far the best characterised tracer for quantification of beta cell mass using the GLP-1 receptor as target. The uptake of the tracer in the pancreas correlates linearly with beta cell mass [15–18] and not with alpha cell mass [15], and remains unaffected by insulitis [16, 18]. Although some expression of the GLP-1 receptor can be found on delta cells [29], islet mass is made up of maximum 5% of delta cells [30, 31], meaning that their influence on tracer uptake will not lead to relevant bias. Furthermore, we have previously shown colocalisation between uptake of radiolabelled exendin and insulin-positive regions in human pancreas tissue, with a distinctly higher uptake compared with the background activity in the exocrine tissue (unpublished data: M. Gotthardt, T.J.P. Jansen, M. Buitinga, C. Frielink, M.W.J. Stommel, M.B. van der Kolk, H. van Goor, B.E. de Galan, M. Boss and M .Brom). An important matter to keep in mind when using exendin PET is the downregulation of GLP-1 receptor expression with prolonged hyperglycaemia [32, 33], which could affect quantification of beta cell mass [34]. To minimise biased results because of differences in blood glucose values between the two groups, participants were asked to keep their blood glucose in the normal range before PET imaging. Blood glucose levels measured just before image acquisition did not differ between the groups. We would also like to point out that alpha cells can increase intra-islet GLP-1 levels, potentially saturating the GLP-1 receptors on the beta cells leading to lower uptake of our tracer [35, 36]. To minimise this effect, use of dipeptidyl peptidase-4 inhibitors in the past 6 months was an exclusion criterion and all participants had been fasting for at least 4 h before the PET/CT scan.

In addition to measuring beta cell mass, alpha cell mass would also be interesting to include when studying the relation between beta cell mass and glucose variability. Pancreas volume substantially decreases in type 1 diabetes [37], but data obtained from donor pancreases showed that alpha cell mass did not change [30]. It would be valuable to see how alpha cell mass relates to beta cell mass and function in an in vivo setting. The development of imaging techniques that measure alpha cell mass is currently ongoing, the combination of alpha cell mass and beta cell mass imaging might lead to new insights regarding glucose variability. Studies have already been performed with a tracer targeting the glucagon receptor [38, 39], and novel tracers may give us detailed information on the role pancreatic cells have on glycaemic variability.

Our study has strengths and limitations. A strength is the extensive phenotyping of the study participants with regard to both beta cell function and mass, which allowed us to examine the (independent) role of beta cell mass. One of the limitations is that our imaging data on residual beta cells could not be validated by direct histological examination of pancreatic tissue. Acquiring histology data would entail serious risks for the participants and is not feasible for ethical reasons [40]. However, we have previously demonstrated GLP-1 receptor expression in insulin-positive and insulin-negative beta cells of individuals with type 1 diabetes (unpublished data: M. Boss, I. Kusmartseva, W. Woliner-van der Weg, L. Joosten, M. Brom, M. Béhe, C.J. Tack, O.C. Boerman, M.J.R. Janssen, M. Atkinson and M. Gotthardt) and have shown in rodents as well as in humans that radiolabelled exendin is a good biomarker for beta cell mass, demonstrating the high specificity of the tracer [15–18]. Another limitation is the small group size. However, participation in this study required individuals to undergo extensive study procedures that were both labour-intensive and burdensome for participants and research staff. Although this may have influenced the statistical power to demonstrate differences in C-peptide levels between the groups, the number was sufficient for the primary outcome, i.e. the beta cell mass and its association with beneficial glycaemic variables as previously described. Finally, the imbalance of female/male participants between the groups may be a limitation, but there are no indications that sex is related to differences in beta cell function or glycaemic control [41], therefore we expect that our results were not influenced by this imbalance.

In summary, our data show that beta cell mass is distinctly higher in people with type 1 diabetes and relatively stable glucose control compared with people with type 1 diabetes in whom glucose levels are much more variable. This finding might point towards an important role that residual beta cells can play in maintaining glycaemic stability and underscores the importance of keeping viable beta cells, even if they appear ‘non-functional’. Surviving beta cells are also of great interest for novel and future interventions that may help to restore or expand their functionality and improve glycaemic control. Exendin PET may contribute to detect these residual beta cells and also represents a valuable tool for clinical studies longitudinally monitoring beta cell mass during the course of diabetes or interventions aiming at preserving beta cell mass in clinical studies.

## Data Availability

The datasets generated during and/or analysed during the current study are available from the corresponding author on reasonable request.

## Abbreviations

CGM: Continuous glucose monitoring
CT: Computed tomography
GLP-1: Glucagon-like peptide 1
HGV: High glucose variability
LGV: Low glucose variability
MMTT: Mixed-meal tolerance test
PET: Positron emission tomography
SUVmean: Mean standardised uptake value
TAR: Time above range
TBR: Time below range
TIR: Time in range
VOI: Volume of interest
